# 

**Published:** 1905-01

**Authors:** 


VOL. XX. JANUARY, 1905. No. 7.

INDEXED

### TEXAS Medical Journal.

SUBSCRIPTION, $1.00 A YEAR, IN ADVANCE.

SINGLE COPIES, 10 CENTS.

PUBLISHED AT AUSTIN, TEXAS, BY
F. E. DANIEL, M. D.,
PROPRIETOR.

PRINTED BY THE VON BOECKMANN-JONES CO.

Entered at the Postoffice at Austin, Texas, as Second-class Mail Matter.

